# Individual Differences and Hemispheric Asymmetries for Language and Spatial Attention

**DOI:** 10.3389/fnhum.2018.00380

**Published:** 2018-10-04

**Authors:** Louise O’Regan, Deborah J. Serrien

**Affiliations:** School of Psychology, University of Nottingham, Nottingham, United Kingdom

**Keywords:** laterality, visual half-field, landmark task, handedness, word comprehension

## Abstract

Language and spatial processing are cognitive functions that are asymmetrically distributed across both cerebral hemispheres. In the present study, we compare left- and right-handers on word comprehension using a divided visual field paradigm and spatial attention using a landmark task. We investigate hemispheric asymmetries by assessing the participants’ behavioral metrics; response accuracy, reaction time and their laterality index. The data showed that right-handers benefitted more from left-hemispheric lateralization for language comprehension and right-hemispheric lateralization for spatial attention than left-handers. Furthermore, left-handers demonstrated a more variable distribution across both hemispheres, supporting a less focal profile of functional brain organization. Taken together, the results underline that handedness distinctively modulates hemispheric processing and behavioral performance during verbal and nonverbal tasks. In particular, typical lateralization is most prevalent for right-handers whereas atypical lateralization is more evident for left-handers. These insights contribute to the understanding of individual variation of brain asymmetries and the mechanisms related to changes in cerebral dominance.

## Introduction

The cerebral hemispheres of the human brain have unique properties of information processing; an asymmetry labeled as hemispheric lateralization that implies that cognitive functions are differentially represented in the brain (Josse and Tzourio-Mazoyer, 2004; Vallortigara and Rogers, 2005). The most commonly studied lateralized functions are language and spatial functions, which display respectively left-hemispheric and right-hemispheric superiority. Initial evidence for dominant language processing within the left hemisphere was provided by Broca (1861) and Wernicke (1874), followed by experimental and clinical research that confirmed that language production and comprehension generally rely more heavily on the left than right hemisphere (Springer et al., 1999; Szaflarski et al., 2002). Moreover, language production and aspects of semantic processing are processed within the anterior left hemisphere, including the inferior frontal gyrus, whereas language comprehension is regulated within the posterior temporo-parietal region of the left hemisphere (Price, 2000). In contrast to language functions, spatial processing as required for attention or orientation predominantly activates the fronto-parietal areas of the right hemisphere (Marshall and Fink, 2001). However, right-sided lateralization of spatial abilities has been observed to be less consistent than left-sided lateralization of language processing, which may be due to differences in the intrinsic organization of both systems (Seydell-Greenwald et al., 2014).

In addition to research on the lateralization of both functions, their relationship has also been explored. Complementarity of function implies that localization of function in one hemisphere predicts the processing of another function in the opposite hemisphere (Bryden et al., 1983). Currently, statistical and causal hypotheses exist in the literature and there is evidence for both. According to the statistical hypothesis, the influences that underlie lateralization of complementary functions are independent of one another. That is, although typical asymmetrical profiles (i.e., language to the left side, spatial attention to the right side) occur, these may represent influences that arise from independent sources (Andresen and Marsolek, 2005). Thus, this hypothesis predicts no correlation between both functions (Groen et al., 2012). According to the causal hypothesis, the asymmetrical lateralization of one function forces the other function to the opposing hemisphere. As this premise expects that two complementary functions are opposite in lateralization profile, a negative correlation is predicted (Cai et al., 2013).

Although language and spatial functions are typically lateralised to opposite hemispheres, the asymmetry is reversed or negated in a minority of people (Knecht et al., 2000), which points to flexibility with which the brain regulates cognitive functions. In this respect, factors that influence lateralization patterns inform us about the extent of this flexibility and in addition about implications for the recovery from neural damage. One such factor is handedness, which represents an expression of hemispheric asymmetry for hand movement control, and the preference to use one hand over the other for performing manual tasks (Annett, 2002). Approximately 90% of humans are right-handed whereas 10% are left-handed; a motor asymmetry that has been relatively stable throughout history and across cultures (Coren and Porac, 1977; Annett, 2002). In this respect, handedness selectively regulates the neural processing mechanisms of the motor system (Klöppel et al., 2007; Martin et al., 2011; Reid and Serrien, 2012; Pool et al., 2014; Serrien and Sovijärvi-Spapé, 2016). Furthermore, left-hemispheric language lateralization associates with handedness in 90%–95% of right-handers and 70%–85% of left-handers (Pujol et al., 1999; Knecht et al., 2000; Flöel et al., 2005; Perlaki et al., 2013; Mazoyer et al., 2014), indicating higher variation due to left-handedness. In addition, Knecht et al. (2000) found that atypical right-hemispheric language lateralization increased from 4% in consistent right-handers to 27% in consistent left-handers. Overall, the research illustrates that more atypical language lateralization occurs due to non-right-handedness (Tzourio et al., 1998). In contrast to language studies, the association between spatial lateralization and influence of handedness has been less explored and evidence is inconsistent. Whereas some work shows that right-hemispheric spatial dominance is fairly evenly represented in right- and left-handers, albeit with different degrees of occurrence (Flöel et al., 2005; Powell et al., 2012), other reports have revealed that the right hemisphere controls spatial tasks in right-handers whereas there is no hemispheric preference in left-handers (Vogel et al., 2003).

The literature indicates that the relationship between hemispheric asymmetries of cognitive traits and handedness is not well-defined. This is especially the case for spatial functions that have not been extensively studied, possibly due to their weaker lateralization patterns as compared to language-mediated asymmetries. Therefore, an improved understanding of the relationship will help to clarify variations of hemispheric lateralization patterns. In this respect, handedness may be a useful tool to test the flexibility of brain organization. In the present work, we study hemispheric asymmetries and complementarity of function by examining language comprehension and spatial attention in a group of left- and right-handers. We use experimental paradigms that reliably measure stimulus processing in both cerebral hemispheres. In this study, we hypothesize that left- and right-handers have distinct lateralization profiles for language and spatial processing due to different characteristics of hemispheric organization in these groups.

## Materials and Methods

### Participants

A total of 46 individuals participated in this study (*M*_AGE_ = 22.7 ± 1.0). They reported no history of neurological or psychiatric illnesses as evaluated by a standardized questionnaire, and had normal or corrected-to-normal vision. Participants gave written consent prior to the start of the experiment in accordance with the Declaration of Helsinki. The study was approved by the School of Psychology Ethics Committee, University of Nottingham.

### Handedness Questionnaire

All participants completed a handedness questionnaire that measured hand preference for unimanual and bimanual manipulation tasks and that consisted of 18 items (i.e., write, use spoon to stir, hold toothbrush, throw ball, hold racquet, thread a needle, pour water from jug, put key in keyhole, draw, carry full glass of water, use computer mouse, hold hammer, open drawer, unscrew lid from jar, strike match to light, hold knife to cut, broom sweeping, peel apple). We also examined the hand preference of communication activities and used questions that involved gestures and that consisted of seven items (time out sign, point, wave, count on fingers, thumbs up, folded arms, raise finger to lips). Both types of questionnaire items reflect self-reports of manual preferences that involve primarily a cognitive component such as memory (Nalçaci et al., 2001).

### Experimental Tasks

#### Language Processing

##### Aim

Language comprehension and conceptual processing of word forms is strongly driven by the left hemisphere (Price, 2000). In this study, we contrast the processing of concrete action words that relate to hand activities (e.g., pinch, clap) and abstract emotion words that denote internal states (e.g., wish, trust) based on the premise that these word meanings are acquired through interaction with the action, object or process that is represented by the word (Hauk et al., 2004). We examine word comprehension using the divided visual field paradigm, which permits to selectively bias hemispheric processing. Although this technique provides indirect behavioral measures of the brain’s processing abilities, they can be used as robust and reliable predictors of lateralization patterns (Hunter and Brysbaert, 2008).

##### Procedure

**Figure 1:**
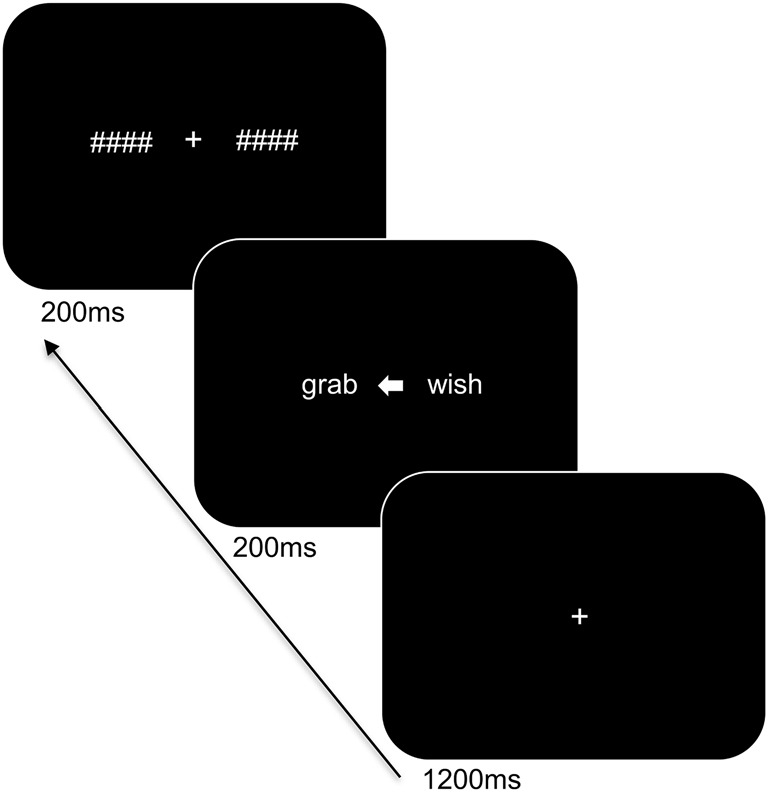
Trial sequence and timing of the language task. After presentation of the fixation cross, two words were shown bilaterally, with an arrowhead pointing to the target word. The words were subsequently replaced by backward masks followed by an intertrial interval.

Participants were seated at a viewing distance of 70 cm from a computer monitor with head rested on a chinrest. The trial sequence of the task is displayed in Figure 1 and was presented using PsychoPy (Peirce, 2007). Stimuli were in white Arial font on a black background and subtended 1.1° of visual angle in height. The trial sequence consisted of a centrally presented fixation cross for 1,200 ms followed by a white arrow for 200 ms alongside two words: one word on the left and one on the right side of the arrow. The words involved a target word and a distractor word of a different category (i.e., an action word and an emotion word) and were matched for length (4–5 letters). The arrowhead pointed either to the left (<) or to the right (>) to indicate the target word and to ensure that participants focused at central fixation. The direction of presentation of the arrow varied pseudo-randomly. The presented words were replaced by backward masks that matched the words for length and remained on the screen for 200 ms followed by an inter-stimulus interval of 1,000 ms. The participants were instructed to indicate the category of the target word by means of a bimanual response (i.e., pressing two keys simultaneously with both index or middle fingers to refer to one or the other category) and to respond as accurately and as quickly as possible. A total of 192 trials was presented. Practice trials were provided to the participants to familiarize them with the task demands and breaks were offered during the experiment.

#### Spatial Processing

##### Aim

Spatial attention is a cognitive function that is strongly mediated by the right hemisphere in the general population (Vogel et al., 2003). This distribution typically leads to attentional asymmetries towards the left side of space known as pseudoneglect (Bowers and Heilman, 1980), which can be observed experimentally when performing the landmark task (i.e., judging whether or not a presented line is correctly bisected) and the line bisection task (i.e., indicating the center of a presented line). Due to the distinct demands of the tasks, both involve different strategies and neural networks (Cavézian et al., 2012).

##### Procedure Landmark Task

**Figure 2:**
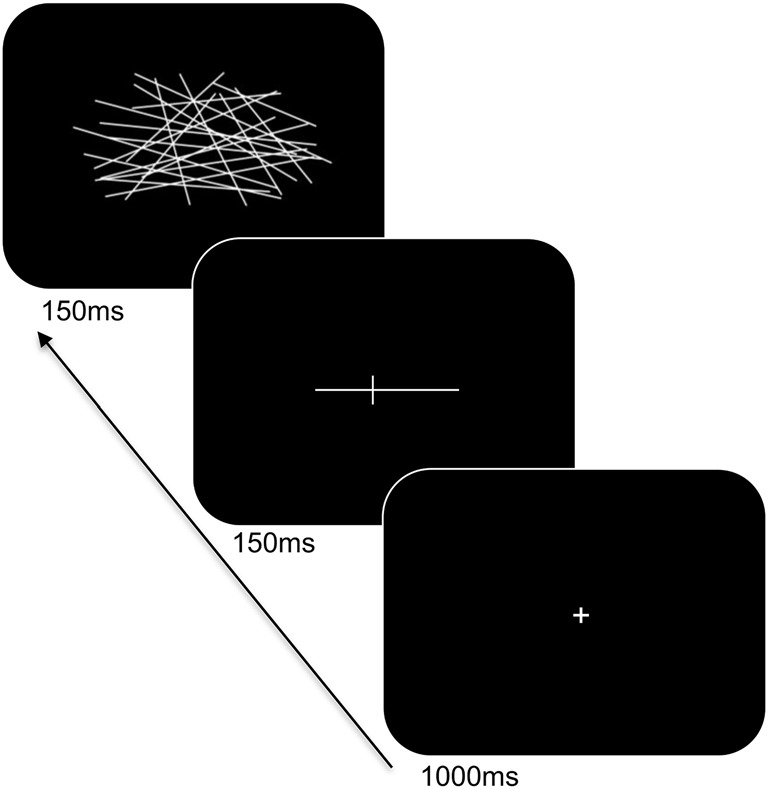
Trial sequence and timing of the landmark task. After presentation of the fixation cross, a pre-marked horizontal line was shown for 150 ms. Following a delay, the line was backward-masked with a series of criss-crossed lines followed by an intertrial interval.

Participants were seated at a viewing distance of 70 cm from a computer monitor with head rested on a chinrest. A computerized version of the landmark task was used and its trial sequence is shown in Figure 2. For each trial, a fixation cross appeared for 1,000 ms followed by a horizontal white line at the center of the screen. The horizontal lines consisted of different lengths (12 cm and 2 cm), were pre-marked with a small transection line, and were presented for 150 ms. The long line subtended 9.74° × 0.04° of visual angle and the short line subtended 1.65° × 0.04° of visual angle. The position of the transection line varied such that the marker randomly appeared at positions of ±7.5% or ±5% of the absolute length of the horizontal line from the true midpoint. Following a delay of 150 ms, the lines were backward-masked with a series of criss-crossed lines for 150 ms. The horizontal lines were incorrectly bisected either to the left or right of the true midpoint on 60% of the trials and correctly bisected on 40% of the trials (control trials). The participants were told to respond using their index finger if the line was correctly bisected, or, middle finger if the line was incorrectly bisected. Participants responded using the left or right hand (counterbalanced across blocks) and to respond as accurately and as quickly as possible. There was a total of 160 trials. Practice trials were provided to the participants to familiarize with the task demands and opportunities were offered to take breaks during the experiment.

##### Procedure Line Bisection Task

A pen and paper line bisection task was used. The task consisted of horizontal black lines ranging 2–20 cm and with a thickness of 2 mm presented in the center of a white A4-sized sheet of paper. The participants were asked to bisect the lines into two parts of equal length using their left or right hand. The task was conducted three times with each hand.

### Measurements and Statistical Analyses

The participants’ data of the handedness questionnaire and experimental tasks were collected and analyzed accordingly through mixed-design analysis of variances (ANOVAs), frequency and correlation analyses. Mean ± SE are reported in the “Results” section.

#### Handedness Questionnaire

The handedness questionnaire provided a separate score for the manipulation actions and for the gestures. The questionnaire used a 5-point Likert scale that ranged between always left, usually left, equal, usually right and always right. According to this format, the score per item was calculated with a value of 0 to always left, 1 to usually left, 2 to both equally, 3 to usually right and a value of 4 to always right. Thereafter, the scores of the items were added for each participant, and divided by the maximum score of the questionnaire, and multiplied by 100. This provided a handedness score that ranged from 0 (extreme left-handedness) to 100 (extreme right-handedness) with 50 (ambidextrous) as intermediate value. The handedness score of the manipulation activities was used to classify the participants into a group of 21 left-handers (*M*_AGE_ = 22.8 ± 1.5; *M*_HAND_ = 21.2 ± 2.6) and 25 right-handers (*M*_AGE_ = 22.6 ± 0.5; *M*_HAND_ = 85.4 ± 1.8) with values <50 indicating a left-hander and values >50 specifying a right-hander. Writing hand was also a condition for handedness as most people are not able to learn and perform writing equally well with either hand, and most individuals will categorize their handedness based on their writing hand (Perelle and Ehrman, 2005). All participants met both the criteria of handedness score and writing hand. The family history of left-handedness of the participants was also established and provided six cases for the left-handed group (28.5%) and three cases for the right-handed group (12.0%) with one or both parents being a left-hander, displaying an inheritance component.

The handedness score of the manipulation actions and gestures were analyzed using a mixed 2 × 2 ANOVA (Handedness Group: left- vs. right-handers × Action Type: manipulation vs. gestures). The between-subject factor was Handedness Group whereas the within-subject factor was Action Type.

#### Language and Spatial Attention Tasks

The measurements of the language and landmark tasks included response accuracy and reaction time. We further calculated a laterality index for both measurements, providing a quantification of hemispheric asymmetry, with the sign indicating the direction of the bias. The laterality index for response accuracy (LI_ACC_) used the formula [R − L]/[R + L] × 100, where R and L reflect the percentage of correct responses for stimuli presented in the right and left visual field, respectively. The laterality index for reaction time (LI_TIM_) used the formula [L − R]/[L + R] × 100 where R and L represent the reaction times for correct trials with stimuli presented in the right and left visual field, respectively. Positive scores of LI_ACC_ and LI_TIM_ indicated a right visual field and hence left hemisphere advantage, whereas negative scores of LI_ACC_ and LI_TIM_ referred to a left visual field and therefore right hemisphere advantage. We further calculated a threshold of the LI (LI_TH_) to measure lateralization of language and spatial attention. In particular, based on the mean and SE of the population sample, the LI_TH_ was set according to the formula: mean-SE if mean >0, and, mean + SE if mean < 0. Subsequently, we quantified lateralization of language and spatial attention for both handedness groups by adopting the formula: LI > LI_TH_ equals left hemisphere dominance; LI <- LI_TH_ equals right hemisphere dominance; |LI| ≤ LI_TH_ equals bilateral dominance. Finally, for the line bisection task, the measurement of interest was the spatial deviation from the true midpoint in millimetres (mm), with negative scores corresponding to a leftward spatial error and positive scores referring to a rightward spatial error.

##### Language Task

The response accuracy scores and reaction times (averaged bimanual responses) were analyzed using mixed 2 × 2 × 2 ANOVAs (Handedness Group: left- vs. right-handers; Visual Field: left vs. right; Word Category: action vs. emotion). The between-subject factor was Handedness Group whereas the within-subject factors were Visual Field and Word Category. LI_ACC_ and LI_TIM_ were analyzed using mixed 2 × 2 ANOVAs (Handedness Group × Word Category). The between-subject factor was Handedness Group whereas the within-subject factor was Word Category.

##### Spatial Attention Landmark Task

The response accuracy scores and reaction times (averaged for transector markers) were analyzed using mixed 2 × 2 × 2 ANOVAs (Handedness: left- vs. right-handers; Marker Position: left vs. right; Line Length: 12 cm vs. 2 cm). The between-subject factor was Handedness Group whereas the within-subject factors were Marker Position and Line Length. LI_ACC_ and LI_TIM_ were analyzed using mixed 2 × 2 ANOVAs (Handedness Group × Line length). The between-subject factor was Handedness Group whereas the within-subject factor was Line Length. In addition, the response accuracy scores and reaction times of the control trials (correctly bisected lines) were analyzed using mixed 2 × 2 ANOVAs (Handedness: left- vs. right-handers; Line Length: 12 cm vs. 2 cm). The between-subject factor was Handedness Group whereas the within-subject factor was Line Length.

##### Spatial Attention Line Bisection Task

The spatial error scores were analyzed using a mixed 2 × 2 × 5 ANOVA (Handedness: left- vs. right-handers; Hand: left vs. right; Line Length; 20 cm vs. 18 cm vs. 12 cm vs. 5 cm vs. 2 cm). The between-subject factor was Handedness Group whereas the within-subject factor was Line Length.

##### Correlation Analyses

We conducted two main types of associations: (1) Spearman’s correlations between LI_ACC_ and LI_TIM_ of the language and spatial attention (landmark) tasks with the handedness scores (manipulation) to evaluate the impact of hand preference on behavior; (2) Pearson’s correlations between the LI_ACC_ and LI_TIM_ of the language and spatial attention (landmark) tasks to investigate whether they share computational characteristics. Significant correlations would suggest that: (1) handedness influences lateralization patterns; and (2) lateralization of both functions associate with one another.

##### Frequency Analyses

We conducted two sets of comparisons: (1) McNemar chi-square tests of the summed LI_TIM_ (language) and LI_ACC_ (landmark) frequencies for each handedness group and category; (2) one-sample proportion tests against the null hypothesis (25%) of the percentage scores of the LI_TIM_-LI_TIM_ quadrants. Significant effects would propose that: (1) handedness promotes the involvement of the left and right hemisphere for language and spatial attention, respectively; (2) the prevalence of (a)typical lateralization varies with handedness.

## Results

### Handedness Questionnaire

**Figure 3:**
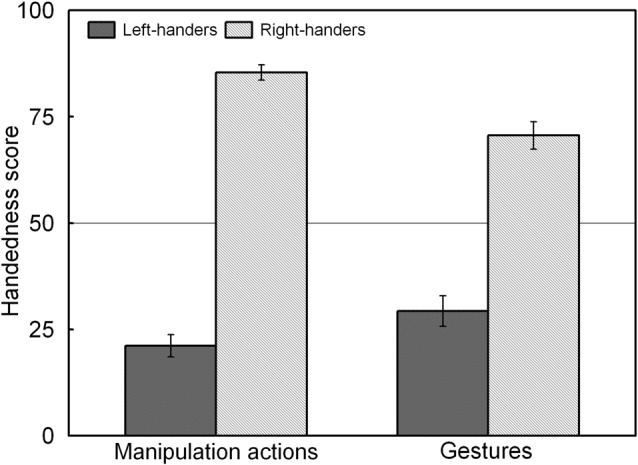
Handedness scores of the manipulation actions and gestures for the left- and right-handers. Both handedness groups show increased use of the non-dominant hand for gestures than for manipulation actions.

The analysis revealed a significant main effect of Handedness Group, *F*_(1,44)_ = 225.92, *p* < 0.05, $\eta_{\text{p}}^{2}$ = 0.836, and a significant Handedness Group × Action Type interaction, *F*_(1,44)_ = 34.45, *p* < 0.05, $\eta_{\text{p}}^{2}$ = 0.439 which is illustrated in Figure 3. The interaction shows that for both groups, the handedness score of the manipulation actions was biased towards their dominant hand whereas the handedness score of the gestures showed increased use of the non-dominant hand (*p* < 0.05 for both left- and right-handers). A correlation analysis between the participants’ handedness scores of the manipulation actions and the gestures showed a positive association (*r*_S(44)_ = 0.86, *p* < 0.05).

### Language Task

#### Response Accuracy

##### Mean Accuracy

The analysis showed a significant main effect of Visual Field, *F*_(1,44)_ = 4.42, *p* < 0.05, $\eta_{\text{p}}^{2}$ = 0.093. There was higher accuracy for words presented to the right visual field (82.8 ± 2.2%) as compared to the left visual field (79.3 ± 2.4%). No significant effect of Handedness Group was noted (*p* > 0.05) with similar accuracy of 81.0 ± 3.5% for the left-handers and 81.1 ± 3.2% for the right-handers.

##### LI_ACC_

No significant effects were noted, *p* > 0.05. However, left-hemispheric involvement was observed for both handedness groups (right-handers: 3.1 ± 2.0; left-handers: 1.9 ± 1.8). Based on the population sample (−2.2 ± 1.3) the LI_TH_ value was set to −0.9, and confirmed dominance of the left hemisphere for the left- as well as the right-handers. A correlation analysis between the LI_ACC_ and the participants’ handedness scores revealed no effect, *p* > 0.05.

#### Reaction Time

##### Mean Reaction Time

The analysis demonstrated a significant main effect of Word Category, *F*_(1,44)_ = 36.52, *p* < 0.05, $\eta_{\text{p}}^{2}$ = 0.448. Participants responded faster to action words (1006.9 ± 25.8 ms) than to emotion words (1075.7 ± 24.0 ms). No significant effect of Handedness Group was noted, *p* > 0.05, with similar reaction times for left-handers (1063.4 ± 34.6 ms) and for right-handers (1020.2 ± 34.7 ms).

##### LI_TIM_

**Figure 4:**
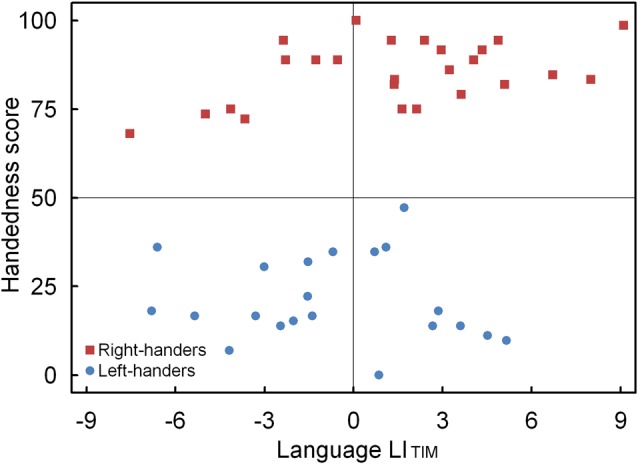
Scatter plot of the LI_TIM_ as a function of the participants’ handedness scores for the language task, with the left- and right-handers demonstrating distinct performance biases.

A significant main effect of Handedness Group was observed, *F*_(1,44)_ = 4.12, *p* < 0.05, $\eta_{\text{p}}^{2}$ = 0.091. The right-handers (1.4 ± 0.8) showed left-hemispheric lateralization as compared to the left-handers (−0.7 ± 0.6). Based on the population sample (0.4 ± 0.6) the LI_TH_ value was set to −0.2, and indicated dominance of the left hemisphere for the right-handers and dominance of the right hemisphere for the left-handers. A scatter plot of the LI_TIM_ for the population sample (*r*_S (44)_ = 0.31, *p* < 0.05) is displayed in Figure 4 and shows the participants who favored the left and right hemisphere for language as well as the number of left- and right-handers in each category. The LI_TIM_ scatter plot illustrates that the majority of the right-handers demonstrated left hemisphere (*N* = 17, 68%) vs. right hemisphere (*N* = 8, 32%) involvement as opposed to the left-handers who depicted a more equivalent pattern between the left hemisphere (*N* = 9, 43%) and the right hemisphere (*N* = 12, 57%).

### Spatial Attention Landmark Task

#### Response Accuracy

##### *Mean Accuracy* (Incorrectly Bisected Lines)

**Figure 5:**
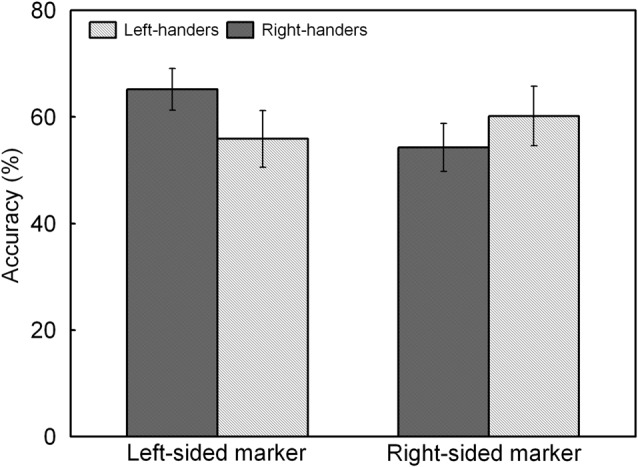
Response accuracy of left- and right-sided marked trials for the left- and right-handers. The right-handers showed higher response accuracy for the trials with marker in the left than right visual field whereas the left-handers did not show differences between both sides.

The analysis indicated a significant main effect of Line Length, *F*_(1,44)_ = 17.60, *p* < 0.05, $\eta_{\text{p}}^{2}$ = 0.286. Accuracy was higher for the long lines (62.3 ± 4.8%) than for the short lines (55.2 ± 4.4%). There was also a significant interaction between Handedness Group and Marker Position, *F*_(1,44)_ = 7.51, *p* < 0.05, $\eta_{\text{p}}^{2}$ = 0.147, which is illustrated in Figure 5. The interaction demonstrates an opposite pattern for both groups, with right-handers obtaining higher accuracy for the trials with marker in the left than right visual field (*p* < 0.05) whereas left-handers did not show differences between both types of trials (*p* > 0.05). No significant effect of Handedness Group was observed with similar accuracy for the left-handers (58.1 ± 4.9%) and the right-handers (59.8 ± 4.2%).

##### LI_ACC_

**Figure 6:**
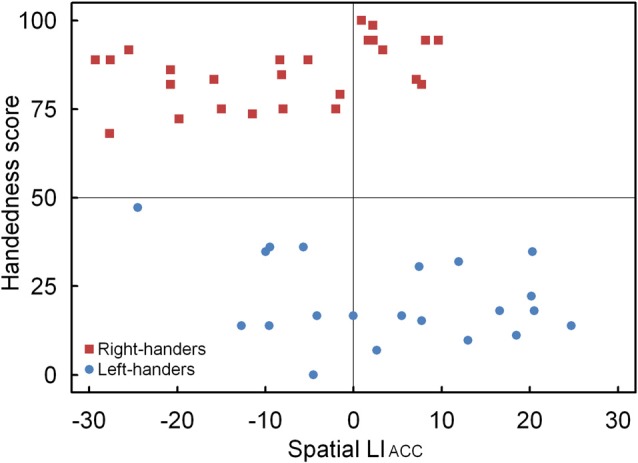
Scatter plot of the LI_ACC_ as a function of the participants’ handedness scores for the landmark task, with the left- and right-handers revealing distinctive performance tendencies.

A significant main effect of Handedness Group was observed, *F*_(1,44)_ = 12.17, *p* < 0.05, $\eta_{\text{p}}^{2}$ = 0.170. The right-handers (−8.2 ± 2.5) showed a right-hemispheric advantage as compared to the left-handers who displayed the opposite bias (4.1 ± 2.9). Based on the population sample (−2.5 ± 2.1), the LI_TH_ value was set to −0.4 which indicated dominance of the right hemisphere for the right-handers and dominance of the left hemisphere for the left-handers. A scatter plot of the LI_ACC_ for the population sample (*r*_S(44)_ = −0.30, *p* < 0.05) is presented in Figure 6 and reveals the participants who favored the left and right hemisphere for spatial attention as well as the number of left- and right-handers in each category. The LI_ACC_ scatter plot shows that the majority of the right-handers demonstrated superiority of the right hemisphere (*N* = 16, 64%) vs. the left hemisphere (*N* = 9, 36%) as opposed to the left-handers who revealed a more balanced profile between the right hemisphere (*N* = 12, 57%) and the left hemisphere (*N* = 9, 43%). On the basis of the LI_TIM_ and LI_ACC_ scatter plots, we evaluated the number of participants with typical lateralization (left-hemispheric dominance for language alongside right-hemispheric dominance for spatial attention) and atypical lateralization (right-hemispheric dominance for language alongside left-hemispheric dominance for spatial attention). McNemar chi-square analysis pointed out that significantly more right-handers showed typical than atypical lateralization, $\chi_{1,N}^{2}$ = 25 = 4.5, *p* < 0.05, whereas no significant difference was observed for left-handers, *p* > 0.05.

##### *Mean Accuracy* (Correctly Bisected Lines)

The analysis indicated no significant effects, *p* > 0.05, with similar accuracy for the left-handers (84.5 ± 3.8%) and the right-handers (86.8 ± 2.0%).

#### Reaction Time

##### *Mean Reaction Time* (Incorrectly Bisected Lines)

The analysis revealed a significant main effect of Marker Position, *F*_(1,44)_ = 10.47, *p* < 0.05, $\eta_{\text{p}}^{2}$ = 0.240. Trials with marker in the left visual field (768.2 ± 9.1 ms) were performed faster than trials with marker in the right visual field (787.5 ± 9.4 ms). There was also a significant main effect of Line Length, *F*_(1,44)_ = 7.28, *p* < 0.05, $\eta_{\text{p}}^{2}$ = 0.189. Reaction times were faster for the long lines (771.3 ± 9.6 ms) than for the short lines (785.2 ± 8.5 ms). No significant effect of Handedness Group was observed, *p* > 0.05, with equivalent times for the left-handers (795.6 ± 12.3 ms) and the right-handers (763.7 ± 12.8 ms).

##### LI_TIM_

There were no significant effects, *p* > 0.05 and similar LI_TIM_ scores were observed for the left-handers (−0.7 ± 0.3) and the right-handers (−1.6 ± 0.6). The correlation analysis between the LI_TIM_ and the participants’ handedness scores indicated no effect, *p* > 0.05.

##### *Mean Reaction Time* (Correctly Bisected Lines)

No significant effects were noted, *p* > 0.05 and similar times were observed for the left-handers (771.7 ± 16.9 ms) and the right-handers (734.2 ± 16.8 ms).

### Spatial Attention Line Bisection Task

#### Spatial Deviation

The analysis revealed a significant main effect of Hand, *F*_(1,44)_ = 4.52, *p* < 0.05, $\eta_{\text{p}}^{2}$ = 0.087. The negative spatial error was higher for the left hand (−1.4 ± 0.4 mm) than the right hand (−0.8 ± 0.3 mm). There was also a significant main effect of Line Length, *F*_(4,176)_ = 12.03, *p* < 0.05, $\eta_{\text{p}}^{2}$ = 0.208. A reduction of the leftward spatial error was noted as the line length became smaller (20 cm: −2.2 ± 0.6; 18 cm: −1.8 ± 0.5; 12 cm: −1.0 ± 0.4; 5 cm: −0.4 ± 0.2; 2 cm: −0.1 ± 0.1).

### Complementary Specialization of Language and Spatial Attention

**Figure 7:**
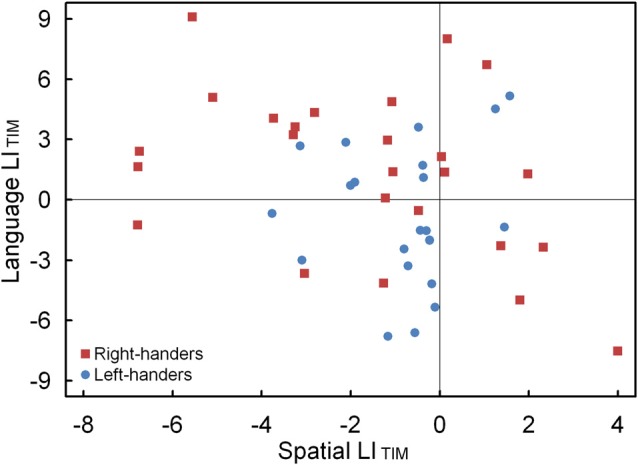
Scatter plot of the LI_TIM_ between the language and landmark tasks, illustrating typical and atypical lateralization patterns for the left- and right-handers.

The LI_ACC_ and LI_TIM_ of the language and landmark tasks were correlated. No significant association was observed for LI_ACC_, *p* > 0.05. Conversely, the LI_TIM_ scores of both tasks revealed a negative correlation for the population sample (*r*_(44)_ = −0.30, *p* < 0.05). The scatter plot is illustrated in Figure 7 and shows the participants with hemispheric dominance for the language and landmark tasks as well as the number of left- and right-handers in each quadrant. The majority of the participants (*N* = 12, 48% right-handers and *N* = 7, 33% left-handers) demonstrated a negative relationship, with language lateralization to the left hemisphere and spatial attention to the right hemisphere, respectively. However, the prevalence of typical lateralization was only significant for the right-handers, *z* = 2.66, *p* < 0.05 [CI: 27.80–68.69], but not for the left-handers, *p* > 0.05. Besides typical lateralization, the scatter plot further illustrates that all the other hemispheric relationships were present. Of note is the combination that involved lateralization of both functions to the right hemisphere for the left-handers (*N* = 11, 52%) as opposed to the right-handers (*N* = 4, 16%). In particular, the prevalence of atypical lateralization of both language and spatial attention to the right hemisphere was significant for the left-handers, *z* = 3.11, *p* < 0.05 [CI: 29.45–73.97], but not for the right-handers, *p* > 0.05. A smaller number of participants showed dominance of both functions to the left hemisphere (right-handers *N* = 5, 20% and left-handers *N* = 2, 10%), or, a reversed lateralization to the opposite hemisphere (right-handers *N* = 4, 16% and left-handers *N* = 1, 5%). No further effects were significant, *p* > 0.05.

## Discussion

In the present work, we studied hemispheric lateralization that captures the dominance of one cerebral hemisphere over the other for information processing and that implies that certain functions are differentially represented in the two sides of the brain (Josse and Tzourio-Mazoyer, 2004; Vallortigara and Rogers, 2005). It is argued that hemispheric lateralization has advantages at the individual level as it increases brain efficiency, and at the population level as it facilitates social coordination and synergistic behaviors between individuals (Corballis, 1989; Ghirlanda and Vallortigara, 2004). Here, we focus on language and spatial attention—two core cognitive functions that underlie many everyday life tasks—and explore the impact of handedness on their regulation. In this respect, handedness is a distinct form of hemispheric specialization for hand movement control, with the left and right hemisphere hosting right- and left-handedness, respectively.

We used experimental paradigms that allowed us to establish indirectly the dominant and non-dominant cerebral hemisphere of language and spatial attention, and employed behavioral metrics of the participants’ decision-making process; reaction time and accuracy (correct responses). That is, reaction time and accuracy were used to quantify the performance dimensions of speed (quickness of problem-solving) and level (degree of problem-solving; Perri et al., 2014). Although, a close coupling between reaction time and accuracy is usually assumed on the basis of similar cognitive and neural processes, this is not always the case as evidenced by our findings and previous work that has demonstrated dissociable influences between both variables as a result of experimental manipulation (van Ede et al., 2012). In particular, it has been argued that attentional cueing involves at least two processes. The first process is preparatory (before the target) and affects both accuracy and reaction time whereas the second process is non-preparatory (after the target) and influences the reaction time only. The distinction implies that changes in accuracy can be explained by an increase of a preparatory signal whereas changes in reaction time are due to an additional component that compares the expected and actual stimulus. This hypothesis suggests that speed and accuracy are processed by closely interacting decision-making systems that couple with specific modulation of neural circuits, i.e., in the supplementary motor area and right prefrontal cortex, respectively (Perri et al., 2014). Besides the computation of the reaction time and accuracy as a result of stimulus presentation to each hemisphere, we also examined the laterality index, which is a valuable measurement as it provides a quantification of the contribution of the dominant hemisphere relative to the non-dominant hemisphere.

### Language Processing and Lateralization Profiles in Left- and Right-Handers

Dominance of the left hemisphere for language-related tasks has been acknowledged since the early observations by Broca (1861) and Wernicke (1874), and subsequently been evidenced through experimental and clinical research (Springer et al., 1999; Price, 2000; Szaflarski et al., 2002). To study language processing, we asked participants to perform a comprehension task that included action and emotion words, presented within a divided visual field paradigm. Using this technique, stimuli presented to the right visual field have preferential access to the processing centers in the dominant left hemisphere of typically lateralised individuals, resulting in a behavioral advantage. Our results revealed that perceived words were responded to more accurately when presented in the right than left visual field, irrespective of word category and handedness group. This finding confirms the efficacy of left-lateralised mechanisms with access to concepts for word understanding, and demonstrates that asymmetries of language processing can be probed with the use of the divided visual field technique (Hunter and Brysbaert, 2008). No other effects with respect to accuracy were noted.

Action words were processed significantly faster than emotion words. This observation provides support for the argument that concrete action words that associate with hand movements are processed more easily than abstract emotion words (Dreyer et al., 2015). Previous research has found that both semantic word categories activate a left-sided network, albeit with distinct functional involvement of multimodal association areas (Binder et al., 2005) and primary/pre- and supplementary motor areas (Moseley et al., 2012; Dreyer et al., 2015). Thus, differences in functional connections between language and motor systems may impact the processing speed of word understanding (Hauk and Pulvermüller, 2004; Boulenger et al., 2006). Alternatively, the right hemisphere may become more directly involved in language functioning with emotional content, affecting the processing demands (Godfrey and Grimshaw, 2016).

Left- and right-handers did not differ in their processing time of the perceived words. However, the LI_TIM_ indicated that the right-handers showed stronger left-hemispheric proficiency than the left-handers. This result suggests increased efficacy for the processing of verbal stimuli in the left than right hemisphere for the right-handers. Conversely, the left-handers showed a hemispheric proficiency pattern that tended towards the right hemisphere, which can be due to a reduced right visual field advantage, or, a lack of processing differences between the visual fields. Furthermore, a correlation analysis between the LI_TIM_ and the participants’ handedness scores showed a positive association, which denotes that hand preference influences the involvement of the dominant hemisphere for word comprehension. Furthermore, the scatter plot indicated that the majority of the right-handers demonstrated the typical profile of left-sided lateralization whereas a more balanced lateralization pattern was noticed across the left-handers. That left-handers have a more equivalent language distribution involving both hemispheres has been highlighted in brain stimulation work (Tussis et al., 2016), suggesting that left-handers have different language functionality than right-handers. Overall, the findings are in line with research that has reported reduced hemispheric asymmetries of language-related areas in left- as compared to right-handers, including a stronger involvement of the right hemisphere (Foundas et al., 1998; Tzourio et al., 1998; Duffau et al., 2008; Li et al., 2015).

### Spatial Processing in the Landmark Task and Distinct Patterns Due to Handedness

Previous studies have shown that spatial location is better identified in the left as compared to right visual field (Durnford and Kimura, 1971; Postma et al., 2006). This effect captures the dominance of the right hemisphere for spatial attention, also labeled as pseudoneglect, and is often explained as a result of an attentional asymmetry of the cerebral hemispheres (Bowers and Heilman, 1980; Kinsbourne, 1993). More recently, pseudoneglect has been explained on the basis of asymmetrical intrahemispheric and interhemispheric connectivity patterns between attention networks (Siman-Tov et al., 2007; Corbetta and Shulman, 2011). In our study, participants performed a landmark task during which they were asked whether presented lines were correctly or incorrectly bisected. Accordingly, this procedure allows the judgment of accurately bisected lines for which both visual fields are recruited as well as inaccurately bisected lines that appear in the left or right visual field at a deviation from the midpoint. Therefore, it enables the participants to choose an appropriate response for both options and ensures that they did not develop a particular performance strategy (Wilkinson and Halligan, 2002). In this respect, the data of the correctly bisected trials revealed no differences between both handedness groups, suggesting that the results of the incorrectly bisected trials can be interpreted accordingly.

Left- and right-handers differed in their accuracy profile as a function of the marker position of the incorrectly bisected lines. In particular, right-handers identified a greater percentage of markers in the left than right visual field as incorrectly deviating from the midpoint. Conversely, left-handers displayed an alternative pattern with accuracy scores that did not differ between both visual fields. Together, these findings denote distinct processing advantages for both handedness groups, with right-handers showing a profile with preferred lateralization to the right hemisphere and left-handers exhibiting a more bilateral pattern with shared functionality. The findings are in line with observations that the right hemisphere controls spatial tasks in right-handers whereas there is no hemispheric superiority in left-handers (Vogel et al., 2003). The results further agree with data that have shown that left-handers have a different neglect-like pattern than right-handers as a result of alertness-related modulations (Bareham et al., 2015). That is, right-handers experience a rightward hemispheric shift in attention with drowsiness whereas left-handers have the opposite pattern. This distinction might be due to the ventral attention network that controls alertness and directs attention to external stimuli, which is mainly right-lateralised in right-handers whereas it is more bilateral or slightly left-lateralised in left-handers (Liu et al., 2009). In this respect, Hécaen and Sauguet (1971) already hinted at a handedness-related variation of brain circuitry when contrasting the visuospatial abilities of patients with left- and right-hemispheric lesions. In particular, they observed that left-handers showed fewer differences than right-handers, suggesting that left-handers have reduced hemispheric dominance, or, a more balanced organization, than right-handers. The discrepancy in the accuracy of the incorrectly bisected lines between both handedness groups was further supported by the LI_ACC_, which revealed that the right-handers demonstrated right-hemispheric lateralization whereas the left-handers tended towards the opposite pattern. In addition, the correlation analysis between the LI_ACC_ and the participants’ handedness scores underlined a negative relationship, proposing that hand preference steers the involvement of the dominant hemisphere for spatial processing. In addition, the scatter plot revealed that the majority of the right-handers demonstrated typical right-hemispheric lateralization whereas a more equivalent profile that involved both hemispheres was noted for the left-handers.

For the reaction time data, no effect of handedness was noted. That is, all participants responded quicker to the marker appearing in the left rather than the right visual field, in line with earlier work (Jewell and McCourt, 2000). In addition, an effect of line length was observed with faster processing for long than short horizontal lines. It has been argued that effects as a function of line length could be related to differences of the salience of the presented stimuli (Benwell et al., 2014) as long lines induce a systematic bias whereas short lines do not (Heber et al., 2010; Benwell et al., 2013). Therefore, the effect of line length could occur as a result of modulation of neural activity, involving a predominant right-sided ventral arousal/re-orienting network for the processing of long lines that becomes less engaged for the processing of short lines (Benwell et al., 2014).

### Spatial Attention and the Line Bisection Task

Dominance of the right hemisphere for spatial attention is characterized by systematic asymmetries towards the left visual field that become evident not only when participants bisect horizontal lines in the landmark task but also when they misplace their subjective midpoint to the left from the body in the line bisection task (Ciçek et al., 2009). As the line bisection task requires the use of the hands, it involves the integration of spatial attention with motor- or handedness-related processing and thus provides a suitable approach to examine how hand dominance influences behavioral performance. However, the line bisection task cannot be used to assess the specific involvement of the right or left side for attentional allocation in space as the spatial error represents an output of interhemispheric collaboration rather than a contribution of one particular hemisphere, as is possible in the landmark task (Cavézian et al., 2012). Therefore, both tasks rely on different cognitive demands and attentional mechanisms, supported by distinct neural networks (Sperber and Karnath, 2016). In particular, the line bisection task activates a bilateral network, including superior parietal and lingual cortex, whereas the landmark task activates a pronounced right hemisphere network, including superior and inferior parietal cortices (Cavézian et al., 2012).

When performing the line bisection task, participants showed a spatial error to the left from the body, which indicates that they overattended to the left hemispace, consistent with an effect of pseudoneglect (Bowers and Heilman, 1980). In particular, they tended to incorrectly bisect the horizontal lines to the left of the center; an asymmetry that guides attention towards the left hemispace (Kinsbourne, 1993). The data further revealed that the left hand generated larger spatial errors than the right hand, independent of handedness group. This disadvantage has also been noted by Brodie and Pettigrew (1996) and could be related to the concurrent regulation of the right hemisphere for left hand movement control and spatial attention. However, we did not observe any specific effects due to handedness group (Scarisbrick et al., 1987; Jewell and McCourt, 2000; Brodie and Dunn, 2005). Finally, we noted an effect of line length, with shorter lines having a smaller bias than long lines, consistent with the functioning of partly different cognitive mechanisms (McCourt and Jewell, 1999).

### Complementary Specialization of Language and Spatial Attention

Left-hemispheric dominance for language and right-hemispheric dominance for spatial attention are present in the general population. This finding has raised the question whether a relationship exists between both asymmetries. In this respect, research has investigated whether the complementary specialization is due to a causal origin (the lateralization of one function causes the opposite lateralization of the other) or represents a statistical phenomenon (different functions lateralize independently). In the case of causal complementarity, a correlation exists between both functions as greater left-hemispheric dominance for language results in greater right-hemispheric dominance for spatial attention, whereas no correlation is evidence of a statistical origin as the functions would be largely statistically independent. Previous studies have provided evidence for both hypotheses (Bryden et al., 1983; Groen et al., 2012; Cai et al., 2013; Zago et al., 2016).

In the present work, we examined the complementary association of both functions at the behavioral level. We observed that the LI_TIM_ scores for language and spatial attention (landmark) negatively correlated with one another for the population sample. This relationship indicates processing efficiency of the respective dominant hemispheres; a pattern that was significant for the right-handers such that stronger left-hemispheric lateralization for language coupled with stronger right-hemispheric lateralization for spatial attention. It suggests that particular characteristics of information processing preferentially operate across hemispheres and cognitive domains. However, the data also showed atypical lateralization patterns, including a combination that involved lateralization of both language and spatial attention to the right hemisphere; a profile that was robustly observed for the left-handers. This observation again points to an increased role of the right hemisphere, resulting in a more dispersed language distribution across the hemispheres, for the left-handers (Tussis et al., 2016). Accordingly, the results highlight that typical lateralization is most prevalent for right-handers whereas atypical lateralization is most apparent for left-handers. More research is required to ascertain the mechanisms that underlie the relationship between lateralization of language and spatial attention as different hemispheric patterns exist across individuals, driven by various factors such as handedness direction and strength (Zago et al., 2016). Together, the findings suggest that although cerebral asymmetries interact and are partly complementary, they depend on multiple influences (Badzakova-Trajkov et al., 2010).

### Handedness and Its Impact on Cognitive Functioning

As is common in the field, the classification of the participants into a left- and a right-handed group was made on the basis of a handedness questionnaire that included a range of manipulation tasks. It is acknowledged that approximately 90% of individuals preferentially use their right hand for complex activities such as object use (e.g., writing with pen), tool use (e.g., hammering) and bimanual coordination (e.g., opening a can). Conversely, the remaining 10% of the population shows an opposite pattern with the left hand being the dominant one or both hands taking on a shared role (Coren and Porac, 1977; Annett, 2002). However, hand asymmetries are not only observed during manipulation tasks as they are also present during communication tasks (gestures; Prieur et al., 2017). Accordingly, our handedness questionnaire comprised gestures such as deictic gestures (e.g., pointing) in order to assess the preferred hand for this type of activity. The data showed that both handedness groups were more consistent in using their dominant hand for manipulation tasks than for gestures. That is, they increasingly used their non-dominant hand for gestures, underlining flexibility in adapting their hand use across contexts.

The study of handedness allows us to better understand the behavioral variance and neural mechanisms of cognitive functioning. In particular, handedness can provide an indication about individual differences in cognitive abilities due to changes in neural capacity, processing speed (Boorman et al., 2007; Roberts et al., 2010) and variation of brain organization (Thiebaut de Schotten et al., 2011; Suchan et al., 2012; Cai et al., 2013; Chechlacz et al., 2015). Our data confirmed individual differences due to handedness as left- and right-handers showed distinctive responses in the language and spatial attention tasks. That is, whereas right-handers showed pronounced hemispheric lateralization for both core functions, left-handers demonstrated reduced lateralization, or, a bilateral distribution across hemispheres, suggesting a less defined organization profile. Therefore, the mechanisms that guide handedness and the underlying brain asymmetries steer the behavioral outcomes as a function of the cognitive function involved (Serrien and Sovijärvi-Spapé, 2013). The present data support the hypothesis that left-handedness underlies between-subject variability for the processing of language and spatial attention tasks, including a less focal or a more distributed pattern. This is in line with the assumption that the two hemispheres operate in a more integrative way with greater interhemispheric communication in left- as compared to right-handers who rely more on the functioning of two independent hemispheres with stronger hemispheric lateralization and asymmetries between hemispheres (Cherbuin and Brinkman, 2006; Serrien et al., 2012). The findings accordingly illustrate individual diversity that associates with a dynamic account of brain organization and underlines the brain’s plasticity for the processing of goal-directed behavior (Serrien et al., 2006).

## Conclusion

Despite the fact that hemispheric lateralization is a natural and fundamental phenomenon, it remains a poorly understood principle of brain organization. This research offers insights into the influence of handedness on the lateralization patterns of two main cognitive functions; language and spatial attention. The results showed that the right-handers benefitted more from left-hemispheric lateralization for language comprehension and right-hemispheric lateralization for spatial attention than the left-handers. Furthermore, the left-handers demonstrated modified lateralization and a bilateral distribution across hemispheres, supporting their more variable profile. Overall, the data indicate that handedness modulates behavioral performance in verbal and nonverbal tasks. In particular, typical lateralization is most prevalent for right-handers whereas atypical lateralization is more evident for left-handers. These insights contribute to the understanding of individual variation of brain asymmetries and the mechanisms related to changes in cerebral dominance.

## Author Contributions

DS: conception of the work, design of the study, data analysis and wrote the manuscript. LR: design of the study, data acquisition, data analysis and reviewed the manuscript.

## Conflict of Interest Statement

The authors declare that the research was conducted in the absence of any commercial or financial relationships that could be construed as a potential conflict of interest.

## References

[B1] Andresen D. R. Marsolek C. J. (2005). Does a causal relation exist between the functional hemispheric asymmetries of visual processing subsystems? Brain Cogn. 59, 135–144. 10.1016/j.bandc.2005.05.010 16157436

[B2] Annett M. (2002). Handedness and Brain Asymmetry: The Right Shift Theory. Hove: Psychology Press, Taylor and Francis.

[B3] Badzakova-Trajkov G. Häberling I. S. Roberts R. P. Corballis M. C. (2010). Cerebral asymmetries: complementary and independent processes. PLoS One 5:e9682. 10.1371/journal.pone.0009682 20300635 PMC2837380

[B4] Bareham C. A. Bekinschtein T. A. Scott S. K. Manly T. (2015). Does left-handedness confer resistance to spatial bias? Sci. Rep. 5:9162. 10.1038/srep09162 25781078 PMC4361991

[B5] Benwell C. S. Harvey M. Gardner S. Thut G. (2013). Stimulus-and state-dependence of systematic bias in spatial attention: additive effects of stimulus-size and time-on-task. Cortex 49, 827–836. 10.1016/j.cortex.2011.12.007 22270326

[B6] Benwell C. S. Harvey M. Thut G. (2014). On the neural origin of pseudoneglect: EEG-correlates of shifts in line bisection performance with manipulation of line length. Neuroimage 86, 370–380. 10.1016/j.neuroimage.2013.10.014 24128738 PMC3980346

[B7] Binder J. R. Westbury C. F. McKiernan K. A. Possing E. T. Medler D. A. (2005). Distinct brain systems for processing concrete and abstract concepts. J. Cogn. Neurosci. 17, 905–917. 10.1162/0898929054021102 16021798

[B8] Boorman E. D. O’Shea J. Sebastian C. Rushworth M. F. Johansen-Berg H. (2007). Individual differences in white-matter microstructure reflect variation in functional connectivity during choice. Curr. Biol. 17, 1426–1431. 10.1016/j.cub.2007.07.040 17689962

[B9] Boulenger V. Roy A. C. Paulignan Y. Deprez V. Jeannerod M. Nazir T. A. (2006). Cross-talk between language processes and overt motor behavior in the first 200 msec of processing. J. Cogn. Neurosci. 18, 1607–1615. 10.1162/jocn.2006.18.10.1607 17014366

[B10] Bowers D. Heilman K. M. (1980). Pseudoneglect: effects of hemispace on a tactile line bisection task. Neuropsychologia 18, 491–498. 10.1016/0028-3932(80)90151-7 6777712

[B11] Broca P. (1861). Sur le principe des localisations cérébrales. Bull. Soc. Anthropol. 2, 190–204.

[B12] Brodie E. E. Dunn E. M. (2005). Visual line bisection in sinistrals and dextrals as a function of hemispace, hand scan direction. Brain Cogn. 58, 149–156. 10.1016/j.bandc.2004.09.019 15919545

[B13] Brodie E. E. Pettigrew L. E. L. (1996). Is left always right? Directional deviations in visual line bisection as a function of hand initial scanning direction. Neuropsychologia 34, 467–470. 10.1016/0028-3932(95)00130-1 8861237

[B14] Bryden M. P. Hécaen H. DeAgostini M. (1983). Patterns of cerebral organization. Brain Lang. 20, 249–262. 10.1016/0093-934x(83)90044-5 6196080

[B15] Cai Q. Van der Haegen L. Brysbaert M. (2013). Complementary hemispheric specialization for language production and visuospatial attention. Proc. Natl. Acad. Sci. U S A 110, E322–E330. 10.1073/pnas.1212956110 23297206 PMC3557046

[B16] Cavézian C. Valadao D. Hurwitz M. Saoud M. Danckert J. (2012). Finding centre: ocular and fMRI investigations of bisection and landmark task performance. Brain Res. 1437, 89–103. 10.1016/j.brainres.2011.12.002 22230669

[B17] Chechlacz M. Gillebert C. R. Vangkilde S. A. Petersen A. Humphreys G. W. (2015). Structural variability within frontoparietal networks and individual differences in attentional functions: an approach using the theory of visual attention. J. Neurosci. 35, 10647–10658. 10.1523/JNEUROSCI.0210-15.2015 26224851 PMC4518045

[B18] Cherbuin N. Brinkman C. (2006). Hemispheric interactions are different in left-handed individuals. Neuropsychology 20, 700–707. 10.1037/0894-4105.20.6.700 17100514

[B19] Ciçek M. Deouell L. Y. Knight R. T. (2009). Brain activity during landmark and line bisection tasks. Front. Hum. Neurosci. 3:7. 10.3389/neuro.09.007.2009 19521543 PMC2694675

[B20] Corballis M. C. (1989). Laterality and human evolution. Psychol. Rev. 96, 492–505. 10.1037/0033-295X.96.3.492 2667014

[B21] Corbetta M. Shulman G. L. (2011). Spatial neglect and attention networks. Annu. Rev. Neurosci. 34, 569–599. 10.1146/annurev-neuro-061010-113731 21692662 PMC3790661

[B22] Coren S. Porac C. (1977). Fifty centuries of right-handedness: the historical record. Science 198, 631–632. 10.1126/science.335510 335510

[B23] Dreyer F. R. Frey D Arana S. von Saldern S. Picht T. Vajkoczy P. Pulvermüller F. (2015). Is the motor system necessary for processing action and abstract emotion words? Evidence from focal brain lesions. Front. Psychol. 6:1661. 10.3389/fpsyg.2015.01661 26617535 PMC4642355

[B24] Duffau H. Leroy M. Gatignol P. (2008). Cortico-subcortical organization of language networks in the right hemisphere: an electrostimulation study in left-handers. Neuropsychologia 46, 3197–31209. 10.1016/j.neuropsychologia.2008.07.017 18708080

[B25] Durnford M. Kimura D. (1971). Right hemisphere specialization for depth perception reflected in visual field differences. Nature 231, 394–395. 10.1038/231394a0 4931009

[B26] Flöel A. Jansen A. Deppe M. Kanowski M. Konrad C. Sommer J.. (2005). Atypical hemispheric dominance for attention: functional MRI topography. J. Cereb. Blood Flow Metab. 25, 1197–1208. 10.1038/sj.jcbfm.9600114 15815582

[B27] Foundas A. L. Eure K. F. Luevano L. F. Weinberger D. R. (1998). MRI asymmetries of Broca’s area: the pars triangularis and pars opercularis. Brain Lang. 64, 282–296. 10.1006/brln.1998.1974 9743543

[B28] Ghirlanda S. Vallortigara G. (2004). The evolution of brain lateralization: a game-theoretical analysis of population structure. Proc. Biol. Sci. 271, 853–857. 10.1098/rspb.2003.2669 15255105 PMC1691668

[B29] Godfrey H. K. Grimshaw G. M. (2016). Emotional language is all right: emotional prosody reduces hemispheric asymmetry for linguistic processing. Laterality 21, 568–584. 10.1080/1357650x.2015.1096940 26508356

[B30] Groen M. A. Whitehouse A. J. Badcock N. A. Bishop D. V. (2012). Does cerebral lateralization develop? A study using functional transcranial Doppler ultrasound assessing lateralization for language production and visuospatial memory. Brain Behav. 2, 256–269. 10.1002/brb3.56 22741100 PMC3381631

[B31] Hauk O. Johnsrude I. Pulvermüller F. (2004). Somatotopic representation of action words in human motor and premotor cortex. Neuron 41, 301–307. 10.1016/s0896-6273(03)00838-9 14741110

[B32] Hauk O. Pulvermüller F. (2004). Neurophysiological distinction of action words in the fronto-central cortex. Hum. Brain Mapp. 21, 191–201. 10.1002/hbm.10157 14755838 PMC6872027

[B33] Heber I. A. Siebertz S. Wolter M. Kuhlen T. Fimm B. (2010). Horizontal and vertical pseudoneglect in peri- and extrapersonal space. Brain Cogn. 73, 160–166. 10.1016/j.bandc.2010.04.006 20537456

[B34] Hécaen H. Sauguet J. (1971). Cerebral dominance in left-handed subjects. Cortex 7, 19–48. 10.1016/s0010-9452(71)80020-5 5567814

[B37] Hunter Z. R. Brysbaert M. (2008). Visual half-field experiments are a good measure of cerebral language dominance if used properly: evidence from fMRI. Neuropsychologia 46, 316–325. 10.1016/j.neuropsychologia.2007.07.007 17716695

[B38] Jewell G. McCourt M. E. (2000). Pseudoneglect: a review and meta-analysis of performance factors in line bisection tasks. Neuropsychologia 38, 93–110. 10.1016/s0028-3932(99)00045-7 10617294

[B39] Josse G. Tzourio-Mazoyer N. (2004). Hemispheric specialization for language. Brain Res. Rev. 44, 1–12. 10.1016/j.brainresrev.2003.10.001 14739000

[B40] Kinsbourne M. (1993). Integrated cortical field model of consciousness. Ciba Found. Symp. 174, 43–50. 8319512 10.1002/9780470514412.ch3

[B41] Klöppel S. van Eimeren T. Glauche V. Vongerichten A. Münchau A. Frackowiak R. S.. (2007). The effect of handedness on cortical motor activation during simple bilateral movements. Neuroimage 34, 274–280. 10.1016/j.neuroimage.2006.08.038 17056278

[B42] Knecht S. Dräger B. Deppe M. Bobe L. Lohmann H. Flöel A.. (2000). Handedness and hemispheric language dominance in healthy humans. Brain 12, 2512–2518. 10.1093/brain/123.12.2512 11099452

[B44] Li M. Chen H. Wang J. Liu F. Wang Y. Lu F.. (2015). Increased cortical thickness and altered functional connectivity of the right superior temporal gyrus in left-handers. Neuropsychologia 67, 27–34. 10.1016/j.neuropsychologia.2014.11.033 25438031

[B45] Liu H. Stufflebeam S. M. Sepulcre J. Hedden T. Buckner R. L. (2009). Evidence from intrinsic activity that asymmetry of the human brain is controlled by multiple factors. Proc. Natl. Acad. Sci. U S A 106, 20499–20503. 10.1073/pnas.0908073106 19918055 PMC2777963

[B46] Marshall J. C. Fink G. R. (2001). Spatial cognition: where we were and where we are. Neuroimage 14, S2–S7. 10.1006/nimg.2001.0834 11373126

[B47] Martin K. Jacobs S. Frey S. H. (2011). Handedness-dependent and -independent cerebral asymmetries in the anterior intraparietal sulcus and ventral premotor cortex during grasp planning. Neuroimage 57, 502–512. 10.1016/j.neuroimage.2011.04.036 21554968 PMC3114104

[B48] Mazoyer B. Zago L. Jobard G. Crivello F. Joliot M. Perchey G.. (2014). Gaussian mixture modeling of hemispheric lateralization for language in a large sample of healthy individuals balanced for handedness. PLoS One 9:e101165. 10.1371/journal.pone.0101165 24977417 PMC4076312

[B49] McCourt M. E. Jewell G. (1999). Visuospatial attention in line bisection: stimulus modulation of pseudoneglect. Neuropsychologia 37, 843–855. 10.1016/s0028-3932(98)00140-7 10408651

[B50] Moseley R. Carota F. Hauk O. Mohr B. Pulvermüller F. (2012). A role for the motor system in binding abstract emotional meaning. Cereb. Cortex 22, 1634–1647. 10.1093/cercor/bhr238 21914634 PMC3377965

[B51] Nalçaci E. Kalaycioğlu C. Çiçek M. Genç Y. (2001). The relationship between handedness and fine motor performance. Cortex 37, 493–500. 10.1016/s0010-9452(08)70589-6 11721861

[B52] Peirce J. W. (2007). PsychoPy-psychophysics software in Python. J. Neurosci. Methods 162, 8–13. 10.1016/j.jneumeth.2006.11.017 17254636 PMC2018741

[B53] Perelle I. B. Ehrman L. (2005). On the other hand. Behav. Genet. 35, 343–350. 10.1007/s10519-005-3226-z 15864449

[B54] Perlaki G. Horvath R. Orsi G. Aradi M. Auer T. Varga E.. (2013). White-matter microstructure and language lateralization in left-handers: a whole-brain MRI analysis. Brain Cogn. 82, 319–328. 10.1016/j.bandc.2013.05.005 23792788

[B55] Perri R. L. Berchicci M. Spinelli D. Di Russo F. (2014). Individual differences in response speed and accuracy are associated to specific brain activities of two interacting systems. Front. Behav. Neurosci. 8:251. 10.3389/fnbeh.2014.00251 25100961 PMC4106455

[B56] Pool E. M. Rehme A. K. Fink G. R. Eickhoff S. B. Grefkens C. (2014). Handedness and effective connectivity of the motor system. Neuroimage 99, 451–460. 10.1016/j.neuroimage.2014.05.048 24862079 PMC4982547

[B57] Postma A. Huntjens R. J. Meuwissen M. Laeng B. (2006). The time course of spatial memory processing in the two hemispheres. Neuropsychologia 44, 1914–1918. 10.1016/j.neuropsychologia.2006.02.001 16530793

[B58] Powell J. L. Kemp G. J. García-Finaña M. (2012). Association between language and spatial laterality and cognitive ability: an fMRI study. Neuroimage 59, 1818–1829. 10.1016/j.neuroimage.2011.08.040 21889594

[B59] Price C. J. (2000). The anatomy of language: contributions from functional neuroimaging. J. Anat. 197, 335–359. 10.1046/j.1469-7580.2000.19730335.x 11117622 PMC1468137

[B60] Prieur J. Barbu S. Blois-Heulin C. (2017). Assessment and analysis of human laterality for manipulation and communication using the Rennes laterality questionnaire. R. Soc. Open Sci. 4:170035. 10.1098/rsos.170035 28878966 PMC5579081

[B61] Pujol J. Deus J. Losilla J. M. Capdevila A. (1999). Cerebral lateralization of language in normal left-handed people studied by functional MRI. Neurology 52, 1038–1043. 10.1212/wnl.52.5.1038 10102425

[B62] Reid C. S. Serrien D. J. (2012). Handedness and the excitability of cortical inhibitory circuits. Behav. Brain Res. 230, 144–148. 10.1016/j.bbr.2012.02.008 22343128

[B63] Roberts R. E. Anderson E. J. Husain M. (2010). Expert cognitive control and individual differences associated with frontal and parietal white matter microstructure. J. Neurosci. 30, 17063–17067. 10.1523/JNEUROSCI.4879-10.2010 21159976 PMC3115511

[B65] Scarisbrick D. J. Tweedy J. R. Kuslansky G. (1987). Hand preference and performance effects on line bisection. Neuropsychologia 25, 695–699. 10.1016/0028-3932(87)90061-3 3658153

[B69] Serrien D. J. Ivry R. B. Swinnen S. P. (2006). Dynamics of hemispheric specialization and integration in the context of motor control. Nat. Rev. Neurosci. 7, 160–166. 10.1038/nrn1849 16429125

[B67] Serrien D. J. Sovijärvi-Spapé M. M. (2013). Cognitive control of response inhibition and switching: hemispheric lateralization and hand preference. Brain Cogn. 82, 283–290. 10.1016/j.bandc.2013.04.013 23742813

[B68] Serrien D. J. Sovijärvi-Spapé M. M. (2016). Manual dexterity: functional lateralisation patterns and motor efficiency. Brain Cogn. 108, 42–46. 10.1016/j.bandc.2016.07.005 27472831

[B66] Serrien D. J. Sovijärvi-Spapé M. M. Farnsworth B. (2012). Bimanual control processes and the role of handedness. Neuropsychology 26, 802–807. 10.1037/a0030154 23106119

[B70] Seydell-Greenwald A. Greenberg A. S. Rauschecker J. P. (2014). Are you listening? Brain activation associated with sustained nonspatial auditory attention in the presence and absence of stimulation. Hum. Brain Mapp. 35, 2233–2252. 10.1002/hbm.22323 23913818 PMC6869372

[B71] Siman-Tov T. Mendelsohn A. Schonberg T. Avidan G. Podlipsky I. Pessoa L.. (2007). Bihemispheric leftward bias in a visuospatial attention-related network. J. Neurosci. 27, 11271–11278. 10.1523/JNEUROSCI.0599-07.2007 17942721 PMC6673032

[B73] Sperber C. Karnath H. O. (2016). Diagnostic validity of line bisection in the acute phase of stroke. Neuropsychologia 82, 200–204. 10.1016/j.neuropsychologia.2016.01.026 26808420

[B74] Springer J. A. Binder J. R. Hammeke T. A. Swanson S. J. Frost J. A. Bellgowan P. S.. (1999). Language dominance in neurologically normal and epilepsy subjects: a functional MRI study. Brain 122, 2033–2046. 10.1093/brain/122.11.2033 10545389

[B75] Suchan J. Rorden C. Karnath H. O. (2012). Neglect severity after left and right brain damage. Neuropsychologia 50, 1136–1141. 10.1016/j.neuropsychologia.2011.12.018 22230231 PMC3348265

[B76] Szaflarski J. P. Binder J. R. Possing E. T. McKiernan K. A. Ward B. D. Hammeke T. A. (2002). Language lateralization in left-handed and ambidextrous people fMRI data. Neurology 59, 238–244. 10.1212/wnl.59.2.238 12136064

[B77] Thiebaut de Schotten M. Dell’Acqua F. Forkel S. J. Simmons A. Vergani F. Murphy D. G.. (2011). A lateralized brain network for visuospatial attention. Nat. Neurosci. 14, 1245–1246. 10.1038/nn.2905 21926985

[B78] Tussis L. Sollmann N. Boeckh-Behrens T. Meyer B. Krieg S. M. (2016). Language function distribution in left-handers: a navigated transcranial magnetic stimulation study. Neuropsychologia 82, 65–73. 10.1016/j.neuropsychologia.2016.01.010 26792365

[B79] Tzourio N. Crivello F. Mellet E. Nkanga-Ngila B. Mazoyer B. (1998). Functional anatomy of dominance for speech comprehension in left handers vs. right handers. Neuroimage 8, 1–16. 10.1006/nimg.1998.0343 9698571

[B80] Vallortigara G. Rogers L. J. (2005). Survival with an asymmetrical brain: advantages and disadvantages of cerebral lateralization. Behav. Brain Sci. 28, 575–589. 10.1017/s0140525x05000105 16209828

[B83] van Ede F. de Lange F. P. Maris E. (2012). Attentional cues affect accuracy and reaction time via different cognitive and neural processes. J. Neurosci. 32, 10408–10412. 10.1523/JNEUROSCI.1337-12.2012 22836273 PMC6703747

[B84] Vogel J. J. Bowers C. A. Vogel D. S. (2003). Cerebral lateralization of spatial abilities: a meta-analysis. Brain Cogn. 52, 197–204. 10.1016/s0278-2626(03)00056-3 12821102

[B85] Wernicke C. (1874). Der Aphasische Symptomencomplex: Eine Psychologische Studie auf Anatomischer Basis. Breslau: Cohn & Weigert.

[B86] Wilkinson D. T. Halligan P. W. (2002). The effects of stimulus symmetry on landmark judgments in left and right visual fields. Neuropsychologia 40, 1045–1058. 10.1016/s0028-3932(01)00142-7 11900756

[B87] Zago L. Petit L. Mellet E. Jobard G. Crivello F. Joliot M.. (2016). The association between hemispheric specialization for language production and for spatial attention depends on left-hand preference strength. Neuropsychologia 93, 394–406. 10.1016/j.neuropsychologia.2015.11.018 26626612

